# Increased serum LOXL2 concentration in pelvic inflammatory disease with pelvic adhesion

**DOI:** 10.1186/s12905-022-01640-1

**Published:** 2022-03-04

**Authors:** Chan Xie, Bixin Tang, Kunlun Wu, Qingyi Meng, Fang Wang

**Affiliations:** Department of TCM Gynecology, Shanghai Pudong New District Gongli Hospital, Second Military Medical University, No.219 Miaopu Road, Pudong New District, Shanghai, 200135 People’s Republic of China

**Keywords:** Pelvic inflammatory disease, Pelvic adhesion, Lysyl oxidase-like 2, Pelvic adhesion

## Abstract

**Background:**

Lysyl oxidase-like 2 (LOXL2) belongs to a family of the LOX secretory enzyme, which involves the cross-linkage of extracellular matrix (ECM) proteins. Here, we aimed to analyze the correlation between serum LOXL2 and pelvic adhesion in chronic pelvic inflammatory disease (PID).

**Methods:**

A total of 143 patients with PID and 130 healthy controls were included in this study. The serum levels of LOXL2 were measured using enzyme-linked immunosorbent assay (ELISA) kits. The patients were divided into non-adhesion group (102 cases) and adhesion group (41 cases).

**Results:**

It was found that the serum level of LOXL2 expression was elevated in PID patients compared with healthy controls, and was elevated in PID patients with pelvic adhesion compared to patients without adhesion. In all PID patients, serum LOXL2 level was positively correlated with matrix metalloproteinases-9 (MMP-9), transforming growth factor-β (TGF-β1), whole blood viscosity (WBV) at low shear rate (LSR), WBV at high shear rate (HSR), and hematocrit (HcT). Multivariate logistic regression analysis showed that serum LOXL2 level was an independent risk factor for pelvic adhesion in PID patients (OR = 1.058; 95% CI = 1.030–1.086, *P* < 0.001).

**Conclusions:**

Serum LOXL2 level not only predicts the presence of PID, but serum LOXL2 concentration is also associated with the presence of pelvic adhesions.

## Background

Pelvic inflammatory disease (PID) is an inflammatory disease caused by infection of microorganisms in the upper genital tract, such as endocervix, endometrium, fallopian tubes and ovaries. PID is characterized by infiltration of neutrophils and T-lymphocytes in the inflammatory lesions and a high neutrophil cell count in blood. PID can be divided into different types based on manifestations, such as endometritis, salpingitis and peritonitis [1]. Chronic infection and inflammation in PID can lead to several sequelae, such as chronic pelvic pain, endometriosis, and ectopic pregnancy [2]. Furthermore, genital tract infections lead to oviduct damage and its healing process involves scarring and adhesion formation, and this pelvic adhesion consequently increases the risk of tubal factor infertility and ectopic pregnancy [3, 4]. Chronic endometritis may promote intrauterine adhesions through enhancing endometrial fibrosis, thus affecting the endometrial receptivity and pregnancy [5]. Early diagnosis of PID is urgently needed for reducing the incidence of PID sequelae. However, due to subtleness and wide variation in the symptoms and signs, the diagnosis of PID is sometimes difficult may require surgery for validation. Early diagnosis and treatment may prevent inflammatory sequelae and infertility in women. Therefore, it is of importance to explore new biomarkers for early diagnosis of PID.

Lysyl oxidase (LOX) family are amine oxidases, and include LOX and four lysyl oxidase-like proteins (LOXL1-4). Lysyl oxidase like-2 (LOXL2) is a monoamine oxidase, and promotes the cross-linking of collagen and elastic fibers by catalyzing the oxidative deamination of lysine and hydroxylysine residues to form peptide aldehydes in collagen and elastin. The covalent cross-linked collagen and elastin fibers interweave into a stable network structure, making them insoluble in water and resistant to non-specific proteolytic enzymes [6]. Therefore, LOXL2 is essential for the formation of fibrous connective tissue and the stability of extracellular matrix (ECM) [7]. Several studies have shown that LOXL2 plays an important role in the fibrosis process of liver, kidney, lung and other organs [8–10]. Moreover, LOXL2 is a secreted protein in blood and serum LOXL2 level is correlated with the degree of organ fibrosis [11]. We speculate that LOXL2 may also play an important role in endometrial fibrosis of PID. However, the expression of LOXL2 in human serum in PID and whether these proteins are related to endometrial fibrosis has not been reported.

This study aimed to determine the concentration of serum LOXL2 protein in PID, and its correlations with pelvic adhesion and other clinical parameters.

## Methods

### Subjects and sample collection

This retrospective study was carried out in chronic PID patients the Department of Obstetrics and Gynecology at Shanghai Gongli Hospital, between July 2018 and December 2019. We selected 130 healthy subjects who received physical examination during the same period with matched age. Blood samples were collected from 143 PID patients and 130 healthy women. PID was diagnosed based on the following manifestations: (1) History: previous history of PID, repeated attacks lasting for more than 6 months. (2) Symptoms: lower abdominal or lumbosacral pain, pain in nature or dull pain, swelling pain or soreness, aggravation of symptoms after roommate or fatigue, which can be accompanied by increased leucorrhea, low fever, and other manifestations. (3) Signs (gynecological examination): limited uterine activity, tenderness, or lifting and swinging pain; thickening and thickening of accessory area, or touching cystic mass, tenderness; thickening and hardening of uterosacral ligament, tenderness. (4) Auxiliary examination: gynecological color Doppler ultrasonography can detect pelvic effusion or pelvic cystic mass. Leucorrhea routine can have cleanliness or abnormal flora [12]. Pelvic adhesion was confirmed by hysteroscopy.

The exclusion criteria were as follows: Breast feeding, pregnant, taking antibiotics, gynecological tumor, and gynecologic operation within 3 weeks. All patients received the same regimens of antibiotics, including at least three days of intravenously injection antibiotics and at least 14 days of oral antibiotics. PID patients were also given oral ibuprofen sustained-release capsule (Fenbid) once a day, one capsule (0.3 g) each time for ten days before menstruation. The demographical and clinical variables were collected from each patient: including age, white blood cell (WBC) and neutrophil counts, serum CRP and TNF-α levels, whole blood viscosity (WBV) at low shear rate (LSR), WBV at high shear rate (HSR), and hematocrit (HcT). WBV was calculated from HcT and total plasma protein (TP) for wall shear stress at LSR (0.5 s^−1^) and HSR (208 s^−1^) according to a validated formula [13]. LSR: WBV (0.5 s^−1^) = (1.89 × HcT) + 3.76 (TP – 78.42). HSR: WBV (208 s^−1^) = (0.12 × HcT) + 0.17 (TP – 2.07). The Ethics Committee approved this study protocol of Gongli Hospital, and informed consent was obtained from all patients. All the experimental protocol for involving humans was by the guidelines of the Declaration of Helsinki.

### ELISA assay

The LOXL2 (cat no. DY2639-05), MMP-9 (cat no. DMP900), and TGF-β1 (cat no. DY240) levels in the serum samples were analyzed by ELISA kits (R&D Systems, Abingdon, UK). The absorbance was measured at 450 nm in a microplate reader, and cytokine levels were quantified by their corresponding standard curve.

### Statistical analysis

Continuous variables were expressed as median (interquartile range), and analyzed using SPSS 20.0 statistical software package (SPPS, Inc., Chicago, IL). ManneWhitney U test was applied to analyze the differences between healthy women and PID patients, or between patients with and without pelvic adhesions ManneWhitney U test. Spearman’s rank correlation was used to analyze the correlation between serum LOXL2 and other continuous variables. Multiple logistic regression analysis was used to determine the effect of serum LOXL2 on pelvic adhesion in PID patients, which was expressed by odds ratio (or) and 95% confidence interval (CI). *P* < 0.05 was used as the standard with statistical significance.

## Results

### Demographic characteristics, clinical characteristics, and cognitive performances

This study included 143 chronic PID patients and 130 age-matched healthy women. Their clinical parameters were listed in Table 1. The age of the PID patients [38 (33–43) year] and healthy women [37 (32–42) year] were matched (*P* = 0.443). Compared to healthy women, PID patients had significantly higher WBC, neutrophil count, CRP and TNF-α levels before treatment (both *P* < 0.001; Table 1). Among the 143 PID patients, 41 patients had pelvic adhesion. The basic characteristics of PID patients with or without pelvic adhesion are shown in Table 2. Compared to patients without pelvic adhesion, patients with pelvic adhesion had significantly higher WBC, neutrophils, serum levels of CRP, TNF-α, MMP-9, TGF-β1, ICAM-1, WBV at LSR, WBV at HSR, and HcT (both *P* < 0.05; Table 2).

**Table 1:** Demographical and clinical data of controls and PID patients

Variables	Control (n = 130)	PID (n = 143)	*P*
Age (year)	37 (32–42)	38 (33–43)	0.264
WBC (/mm^3^)	7290 (6965–7599)	9297 (8668–9815)	< 0.001
Neutrophils (/mm^3^)	4249 (4065–4520)	7323 (7000–7681)	< 0.001
CRP (mg/L)	0.627 (0.569–0.692)	1.94 (1.76–2.11)	< 0.001
TNF-α (pg/mL)	38.7 (36.7–41.1)	83.6 (78.1–88.9)	< 0.001

Manne Whitney U test was performed

*PID* pelvic inflammatory disease, *WBC* white blood cell, *CRP* C-reactive protein, *TNF-α* tumor necrosis factor-α

**Table 2:** Clinical characteristics of the PID patients with and without pelvic adhesion

Variables	Pelvic adhesions (n = 143)		*P*
	No (n = 102)	Yes (n = 41)	
Age (year)	38 (32–42)	40 (35–45)	0.202
WBC (/mm^3^)	9182 (8606–9712)	9626 (8904–10,080)	0.017
Neutrophils (/mm^3^)	7293 (6973–7612)	7540 (7185–8022)	0.033
CRP (mg/L)	1.90 (1.74–2.08)	2.06 (1.81–2.25)	0.014
TNF-α (pg/mL)	82.8 (77.5–87.2)	87.0 (80.1–94.5)	0.012
MMP-9 (ng/mL)	355.8 (295.5–408.9)	390.8 (360.6–434.4)	0.001
TGF-β1 (pg/mL)	15.8 (14.8–16.8)	17.3(16.1–18.8)	< 0.001
ICAM-1 (ng/mL)	69.5 (64.1–73.6)	75.4 (70.8–80.7)	< 0.001
WBV at LSR (0.5 s^−1^)	66.2 (60.4–72.0)	71.6 (66.7–76.1)	0.001
WBV at HSR (208 s^−1^)	18.7 (17.6–20.3)	19.6 (18.5–21.0)	0.006
HcT (%)	43.0 (41.5–44.7)	45.1 (43.1–46.3)	0.001

ManneWhitney U test was performed

*PID* pelvic inflammatory disease, *WBC* white blood cell, *CRP* C-reactive protein, *TNF-α* tumor necrosis factor-α, *MMP-9* matrix metalloproteinases-9, *TGF-β1* transforming growth factor-β, *ICAM-1* intercellular adhesion molecule-1, *WBV* whole blood viscosity, *LSR* low shear rate, *HSR* high shear rate, *HcT* hematocrit

### Serum LOXL2 concentrations were elevated in PID patients

**Fig. 1:**
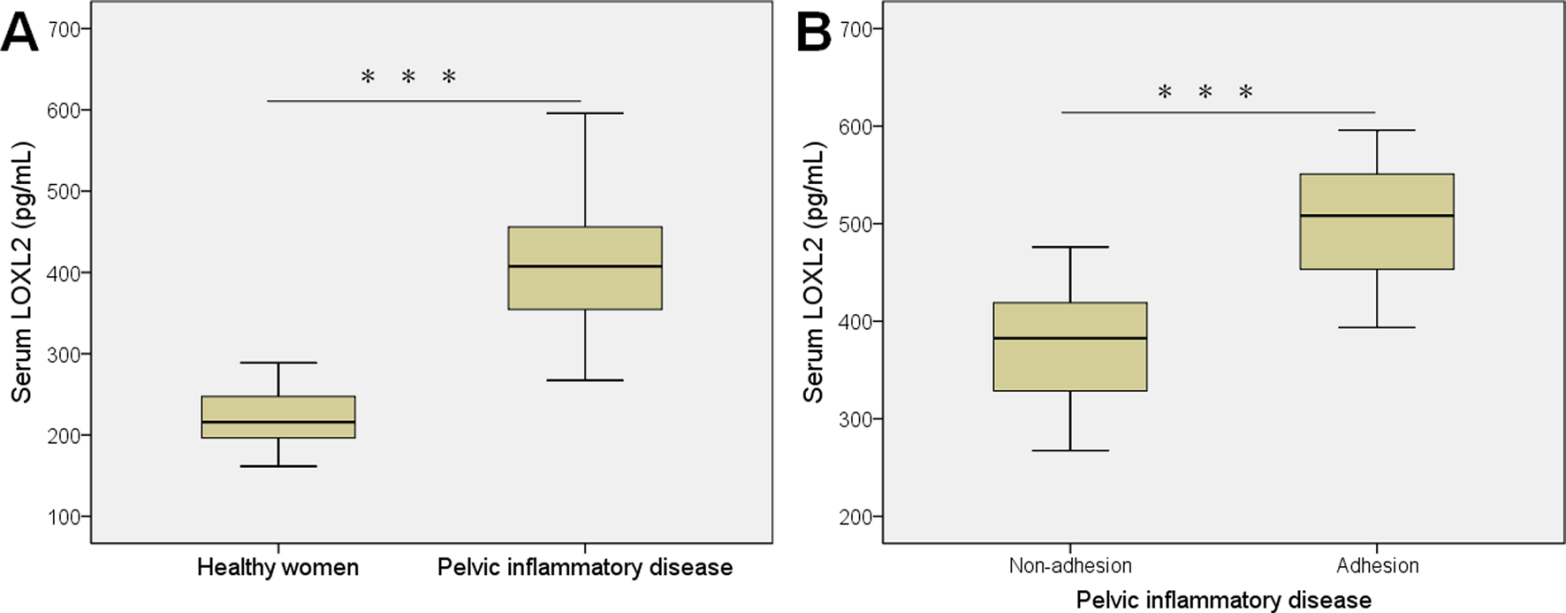
Serum LOXL2 levels in healthy women and PID patients. (**A**) PID patients (n = 143) show higher serum LOXL2 than healthy controls (n = 130) (*P* < 0.001). (**B**) PID patients with pelvic adhesion (n = 41) show higher serum LOXL2 than patients without pelvic adhesion (n = 102). ***P < 0.001

We analyzed the concentration of serum LOXL2 in between the PID patients and healthy women. The serum LOXL2 level was significantly higher in the PID patients [407.4 (351.8–456.2) pg/mL] compared with that in the healthy women [215.9 (196.0–247.4) pg/mL] (*P* < 0.001; Fig. 1A). Moreover, among PID patients, serum LOXL2 level was significantly higher in subjects with pelvic adhesion [508.2 (453.0–554.8) pg/mL] compared to subjects without pelvic adhesion [382.6 (327.5–418.9) pg/mL] (*P* < 0.001; Fig. 1B).

### The correlation between serum LOXL2 with MMP-9, TGF-β1 and ICAM-1 in PID patients

**Fig. 2:**
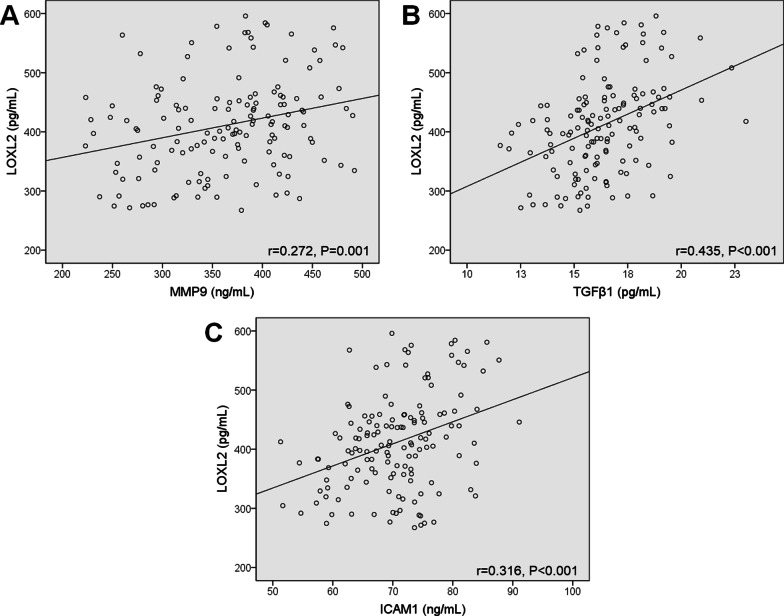
The correlation between serum LOXL2 and other clinical parameters. Serum LOXL2 positive correlates with (**A**) MMP-9 (r = 0.272, *P* = 0.001), (**B**) TGF-β1 (r = 0.435, *P* < 0.001) and (C) ICAM-1 (r = 0.316, *P* < 0.001) in 143 patients with PID. Spearman correlation test was performed. ***P < 0.001

Spearman rank correlation test was applied to investigate the association between serum LOXL2 and pelvic adhesion-related cytokines. Serum LOXL2 correlated positively to MMP-9 (r = 0.272, *P* = 0.001), TGF-β1 (r = 0.435, *P* < 0.001) and ICAM-1 (r = 0.316, *P* < 0.001) in PID patients (Fig. 2A–C).

### Serum LOXL2 was an independent risk factor of pelvic adhesion

In order to explore whether serum LOXL2 is an independent contributor of pelvic adhesion, we performed multivariate logistic regression analyses to calculate the odds ratio (OR) of LOXL2 compared with other risk factors. For each one unit (pg/mL) increase of LOXL2, the adjusted risk of pelvic adhesion increased by 5.8% (OR = 1.058; 95% CI = 1.030–1.086, *P* < 0.001). In addition, WBC, ICAM-1, and WBV at HSR were also associated with the presence of pelvic adhesion in the multivariate regression analysis (Table 3).

**Table 3:** Logistic multivariate regression for new-onset and recurrent AF

Characteristics	Odds ratio	95% confidence interval	*P* value
WBC (/mm^3^)	1.002	1.000–1.003	0.006
ICAM-1 (ng/mL)	1.168	1.032–1.322	0.014
WBV at HSR	1.657	1.017–2.700	0.043
LOXL2 (pg/mL)	1.058	1.030–1.086	< 0.001

Adjust for age, WBC, Neutrophils, CRP, TNF-α, MMP-9, TGF-β1, WBV at LSR, WBV at HSR, and HcT

*WBC* white blood cell, *ICAM-1* intercellular adhesion molecule-1, *WBV* whole blood viscosity, *HSR* high shear rate

## Discussion

In this study, we investigated serum LOXL2 levels in 143 PID patients and 130 age-matched healthy women. Our results showed significantly higher serum LOXL2 level in PID patients compared to age-matched healthy women, and significantly higher serum LOXL2 level in PID patients with pelvic adhesion compared to patients without pelvic adhesion. Serum LOXL2 demonstrated positive correlation with MMP-9, TGF-β1 and ICAM-1 in PID patients. Serum LOXL2, as well as WBC, ICAM-1 and WBV at HSR, is independent risk factor of pelvic adhesion in PID. There LOXL2 may be a good serum biomarker for PID patients with high risk of pelvic adhesion and infertility.

Pelvic Inflammatory Syndrome shares many similar functions with endometriosis, especially considering a pro-inflammatory condition. Accumulating evidence suggests that immune cells, adhesion molecules, extracellular matrix metalloproteinase, and pro-inflammatory cytokines activate/alter the peritoneal microenvironment, creating the conditions for differentiation, adhesion, proliferation, and survival of ectopic endometrial cells [14–16]. The study confirms the hypothesis that LOXL2 is a contributing factor of pelvic adhesion in PID. PID is one common inflammatory disease in reproductive system induced by infections, with sequelae that might affect fertility [3]. Pelvic adhesion is fibrin bands that are caused by PID or surgery [17], with ectopic pregnancy as its major consequence [4]. Adhesion can be aggravated in one type of PID, chronic endometritis, through enhancing fibrosis process [5]. Therefore, the pathological feature of adhesion is fibrosis, and extracellular matrix deposition makes the endometrium replaced by fibrous connective tissue [18]. LOXL2 is involved in fibrogenesis of various diseases, and modulates the process of matrix remodeling and EMT by cross-linking of collagen I [19]. A variety of studies have reported that dysregulation of LOXL2 is strongly associated with cancer progression [20] and fibrosis-related diseases [21]. Moreover, LOXL2 expression was also detected in serums of idiopathic pulmonary fibrosis disease [22], rheumatoid arthritis-associated interstitial lung disease [23] and was positively correlated with atrial fibrosis in patients with atrial fibrillation [24]. This indicates that LOXL2 is a protein secreted from various tissues, and higher serum LOXL2 in PID with pelvic adhesion suggests that LOXL2 might also be highly expressed in endometrium and fallopian tube, which is supported by a study in endometriosis. High LOX expression was observed in endometrial epithelium of patients with endometriosis-associated infertility, and overexpression of LOX increased the expression of genes related to fibrosis and ECM remodeling [25]. PID patients had a higher risk of developing endometriosis [26], and endometriosis is also associated with pelvic adhesion and fibrosis [27, 28]. Therefore, we speculate that LOXL2 may also play an important role in the formation of pelvic adhesion and fibrosis in PID, and more studies are needed to validate this hypothesis in PID tissue and animal model.

A large quantity of serum fibrotic markers has been found as possible biomarkers for PID, and our study showed that serum MMP-9, TGF-β1 and ICAM-1 levels were markedly higher in PID patients with pelvic adhesion. MMP-9 is a member of matrix metalloproteinases (MMPs), and is a key proteinase involved in normal matrix remodeling. MMPs can be produced by inflammatory cells to repair damaged ECM, while excessive MMPs can also promote the ECM degradation [29]. The level of plasma MMP-9 was elevated in PID patients compared with healthy controls [30]. TGF‑β1 signaling pathway is implicated in the fibrosis process, and serum TGF‑β1 level was increased in rats with chronic pelvic inflammatory disease (CPID) [31]. Our study supports this report in PID patients and further found its association with pelvic adhesion. TGF-β has potent fibrotic activity as TGF-β can induce endometrial fibrosis in human endometrial carcinoma cells [32]. ICAM-1 is a adhesion molecule that stimulates leukocyte adhesion. Serum ICAM-1 levels were higher in endometriosis patients compared to healthy women [33]. ICAM-1 can also promote human endometriotic stromal cells proliferation and adhesion [34]. Our previous study showed that ICAM-1 expression in uterus or fallopian tube were markedly increased in PID mice [35]. This study added MMP-9, TGF-β1 and ICAM-1 as new serum biomarkers of PID patients, and further showed their positive correlations with LOXL2. MMP-9 and TGF-β1 are both downstream molecules of LOXL2 in matrix remodeling and fibrogenesis of fibrosis associated diseases [8, 36]. Whether and how LOXL2 modulates MMP-9 and TGF-β1 remain totally unknown in PID and deserves further investigation.

There were some limitations to the study. Firstly, the sample size is small and our findings should be validated in study with larger sample. Secondly, the source of serum LOXL2 in PID patients is still unclear, and should be confirmed in biopsy tissue of PID patients. Thirdly, as an indicator for pelvic adhesion, prospective studies should be conducted to observe the predictive effect of LOXL2 on progression and recurrence of pelvic adhesion. Fourthly, an animal model of PID is needed to elucidate the possible mechanisms of LOXL2 in process of pelvic adhesion and fibrosis.

## Conclusions

Serum LOXL2 levels are increased in PID patients, and also strongly correlated with pelvic adhesion, and fibrosis-related markers. Although these relationship are correlative and not causative, LOXL2 may be a disease-driver in pathogenesis of fibrotic process in PID in animal models. Our study provides LOXL2 as a biomarker for prediction of pelvic adhesion in PID patients.

## Data Availability

Data and materials are available upon request to the corresponding author.

## Abbreviations

ECM: Extracellular matrix
ELISA: Enzyme-linked immunosorbent assay
HcT: Hematocrit
HSR: High shear rate
LOX: Lysyl oxidase
LOXL2: Lysyl oxidase like 2
LSR: Low shear rate
MMP-9: Matrix metalloproteinases-9
PID: Pelvic inflammatory disease
TCM: Traditional Chinese medicine
TGF-β1: Transforming growth factor-β
WBV: Whole blood viscosity
